# Semantic Web Applications and Tools for the Life Sciences: SWAT4LS 2010

**DOI:** 10.1186/1471-2105-13-S1-S1

**Published:** 2012-01-25

**Authors:** Albert Burger, Adrian Paschke, Paolo Romano, M Scott Marshall, Andrea Splendiani

**Affiliations:** 1 Department of Computer Science, Heriot-Watt University & MRC Human Genetics Unit, Edinburgh, UK; 2 Corporate Semantic Web, Freie Universitaet Berlin, Germany; 3 Bioinformatics, IRCCS San Martino University Hospital - IST National Cancer Research Institute, Genova, Italy; 4 Informatics Institute, University of Amsterdam, Amsterdam, The Netherlands; 5 Biomathematics and Bioinformatics Dept., Rothamsted Research, UK

## Abstract

As Semantic Web technologies mature and new releases of key elements, such as SPARQL 1.1 and OWL 2.0, become available, the Life Sciences continue to push the boundaries of these technologies with ever more sophisticated tools and applications. Unsurprisingly, therefore, interest in the SWAT4LS (Semantic Web Applications and Tools for the Life Sciences) activities have remained high, as was evident during the third international SWAT4LS workshop held in Berlin in December 2010. Contributors to this workshop were invited to submit extended versions of their papers, the best of which are now made available in the special supplement of BMC Bioinformatics. The papers reflect the wide range of work in this area, covering the storage and querying of Life Sciences data in RDF triple stores, tools for the development of biomedical ontologies and the semantics-based integration of Life Sciences as well as clinicial data.

## 

Motivated by the ever increasing Semantic Web-based research and development efforts by the Life Sciences community, the SWAT4LS activities started in 2008 with the first of its workshops held in Edinburgh in November 2008. Building on its success, 2009 and 2010 saw equally successful workshop events held in Amsterdam and Berlin, respectively. By now the workshop also offers a set of relevant Semantic Web and Life Sciences tutorials, with the forthcoming SWAT4LS workshop in London in December 2011 comprising of a hackathon day, a tutorial day and the actual workshop day. As co-chairs we are delighted with this growth and hope that the SWAT4LS activities continue to provide an excellent platform for learning and exchange of ideas for the community.

A central component of our efforts are, of course, the special journal issues that are associated with the workshops. This current issue is based on SWAT4LS held in Berlin in December 2010. As always, the papers included in this supplement are significantly revised and extended contributions from the original event and have been subject to a second, independent and rigorous peer review process.

The collection of papers in this special issue discuss research and development in the areas of storage and querying of Life Sciences data in RDF triple stores, a tool to assist with the development of Life Science OWL ontologies and a number of papers on the use of semantic knowledge for the integration of distributed and heterogeneous resources, including widely differing kinds of data, such as about yeast, gene expression patterns and cancer.

As always, endeavours such as SWAT4LS would not succeed without the input from a number of people. In the first instance we would like to thank the authors of the papers included in this supplement. We also thank the reviewers who served on the program committee for the original workshop papers and then again for the papers submitted for this supplement. We are grateful for the sponsorship of SWAT4LS 2010 by Ontotext, Franz Inc, Ontoprise and O'Reilly. Finally, we thank the staff at Biomed Central for their help with editing and processing this supplement.

We hope the readers will find this special supplement useful and look forward to meeting as many of you as possible at future SWAT4LS event. For further details, including links to SWAT4LS workshop proceedings and special journal issues, please see www.swat4ls.org.

Albert Burger, Adrian Paschke, Paolo Romano, M. Scott Marshall and Andrea Splendiani (SWAT4LS Supplement Editors and Co-chairs of SWAT4LS 2010)

